# Prevalence of psychiatric disorders in patients with craniofacial malformations - a statistical analysis

**DOI:** 10.1192/j.eurpsy.2024.1005

**Published:** 2024-08-27

**Authors:** G. Pereira Bernd, V. Dall Agnol Bouvier, T. Brusa da Costa Linn, I. Cho de Almeida, B. de Oliveira de Marchi, L. Guinter Muccillo, C. G. Menezes Chaves Barcellos, C. Paz Portinho, M. V. Martins Collares

**Affiliations:** ^1^Universidade Federal do Rio Grande do Sul, Porto Alegre; ^2^Feevale, Novo Hamburgo, Brazil

## Abstract

**Introduction:**

Craniofacial malformations have long been associated with a heightened risk of psychiatric disorders. Understanding this link is crucial, as it can inform early intervention and support for affected individuals, enhancing their overall well-being. Research in this area aims to shed light on the prevalence and nature of these disorders within the craniofacial population, ultimately improving healthcare and quality of life for affected individuals.

**Objectives:**

This study aims to establish a comprehensive understanding of the relationship between craniofacial malformations and psychiatric disorders. Specifically, our objectives include: assessing prevalence, identifying risk factors, evaluating impact and informing clinical practice. This research aims to improve the holistic care and mental well-being of individuals with craniofacial malformations, contributing to a more comprehensive approach in the field of psychiatry.

**Methods:**

This cross-sectional study was conducted at a prominent referral hospital named Hospital de Clínicas de Porto Alegre during the month of August 2023.

Participant Selection: Patients with craniofacial malformations of all ages and both genders.

Data Collection: We conducted structured interviews with participants to gather demographic information, medical history, and details of their craniofacial conditions.

Medical Records Review: Medical records were reviewed to corroborate craniofacial diagnoses and identify any comorbid conditions.

Statistical Analysis: Data were analyzed using appropriate statistical techniques to assess the association between craniofacial malformations and psychiatric disorders.

Ethical Considerations: The study adhered to all ethical guidelines, with informed consent obtained from participants or their legal guardians. Ethical approval was obtained from the hospital’s Institutional Review Board.

Data Handling: Confidentiality and data security were ensured throughout the study, with all data anonymized to protect participant privacy.

**Results:**

In our study, we assessed 132 different patients, comprising 62 females and 70 males. The youngest patient was 2 months old, while the oldest was 56 years old. The mean age of the patients was 16.22 years, with a median of 9 years, a harmonic mean of 18 years, and a standard deviation of 15.23 years.

Among the patients, 24 exhibited psychiatric disorders, evenly split between 12 males and 12 females. Their average age was 16.21 years, with a median of 10 years, a harmonic mean of 6.13, and a standard deviation of 14.57. The youngest patient with evidence of a psychiatric disorder was 2 years old.

**Image:**

**Figure:**
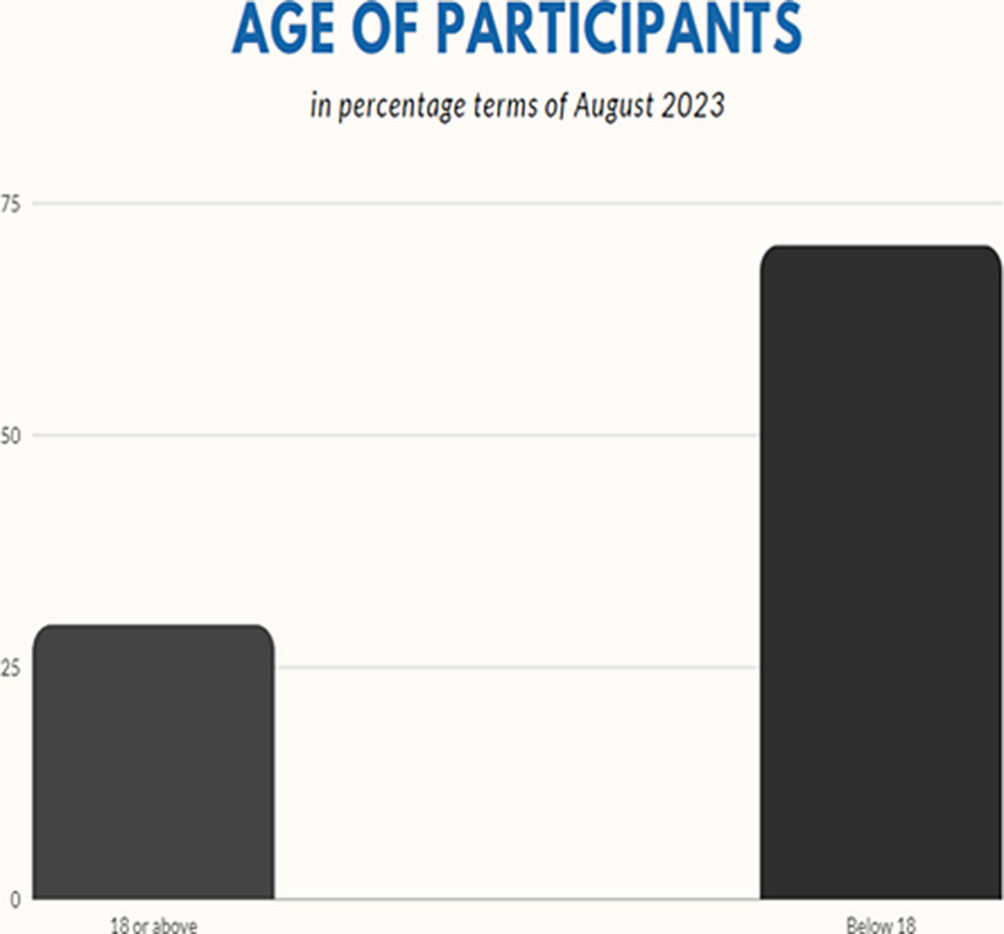

**Image 2:**

**Figure:**
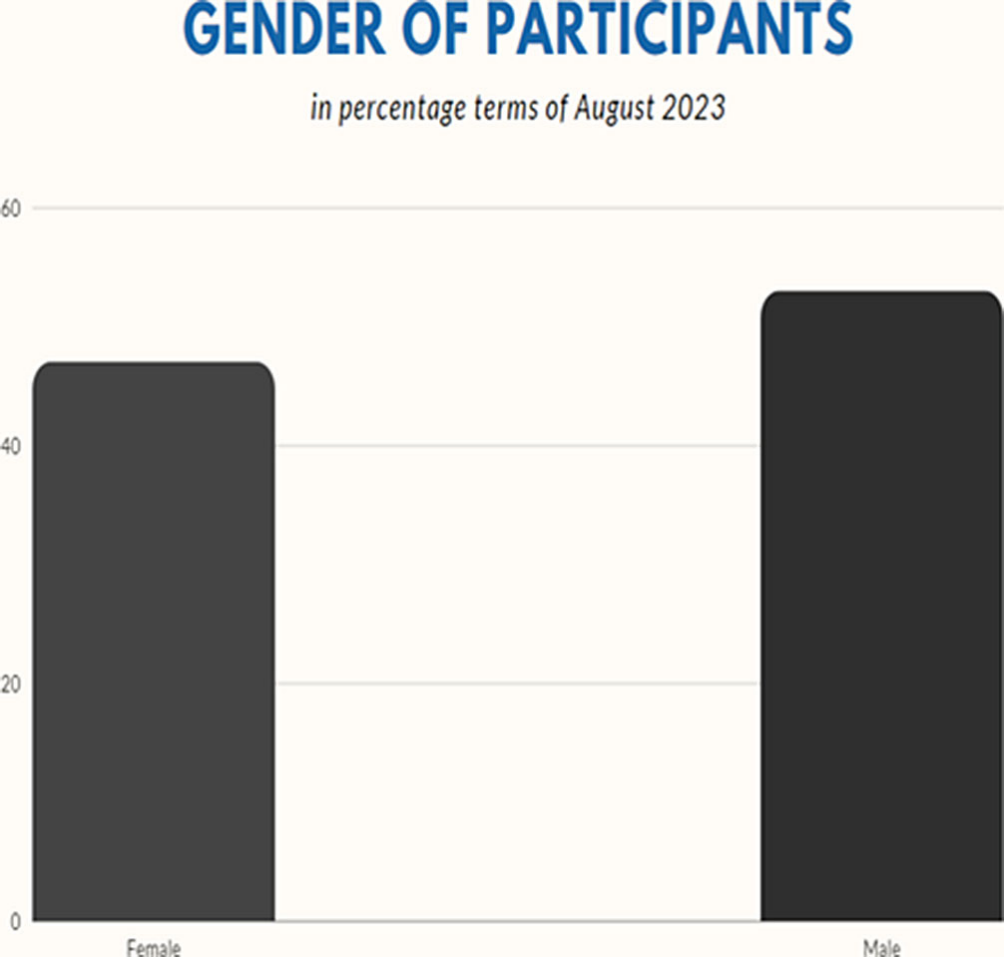

**Conclusions:**

Our study underscores the prevalence of psychiatric disorders among craniofacial patients, which seems to be greater than the general population, emphasizing the need for integrated care that considers both medical and psychological aspects, thus enhancing the overall well-being of these individuals.

**Disclosure of Interest:**

None Declared

